# A crystal glass–nanostructured Al-based electrocatalyst for hydrogen evolution reaction

**DOI:** 10.1126/sciadv.add6421

**Published:** 2022-11-04

**Authors:** Sida Liu, Hongkun Li, Jing Zhong, Kai Xu, Ge Wu, Chang Liu, Binbin Zhou, Yang Yan, Lanxi Li, Wenhao Cha, Keke Chang, Yang Yang Li, Jian Lu

**Affiliations:** ^1^ Centre for Advanced Structural Materials, City University of Hong Kong Shenzhen Research Institute, Greater Bay Joint Division, Shenyang National Laboratory for Materials Science, Shenzhen 518057, China.; ^2^ Hong Kong Branch of National Precious Metals Material Engineering Research Center, City University of Hong Kong, Hong Kong SAR, China.; ^3^ Department of Mechanical Engineering, City University of Hong Kong, Hong Kong SAR, China.; ^4^ Department of Materials Science and Engineering, City University of Hong Kong, Hong Kong SAR, China.; ^5^ Key Laboratory of Marine Materials and Related Technologies, Zhejiang Key Laboratory of Marine Materials and Protective Technologies, Ningbo Institute of Materials Technology and Engineering, Chinese Academy of Sciences, Ningbo, Zhejiang 315201, China.; ^6^ Center for Advancing Materials Performance from the Nanoscale and Hysitron Applied Research Center in China, State Key Laboratory for Mechanical Behavior of Materials, Xi’an Jiaotong University, Xi’an 710049, China.; ^7^ Max-Planck-Institut für Eisenforschung, Max-Planck-Straße 1, Düsseldorf 40237, Germany.; ^8^ Faculty of Georesources and Materials Engineering, RWTH Aachen University, Aachen 52056, Germany.; ^9^ CityU-Shenzhen Futian Research Institute, Shenzhen 518045, China.

## Abstract

Platinum-based catalysts are widely used in hydrogen evolution reactions; however, their applications are restricted because of the cost-efficiency trade-off. Here, we present a thermodynamics-based design strategy for synthesizing an Al_73_Mn_7_Ru_20_ (atomic %) metal catalyst via combinatorial magnetron co-sputtering. The new electrocatalyst is composed of ~2 nanometers of medium-entropy nanocrystals surrounded by ~2 nanometers of amorphous regions. The catalyst exhibits exceptional performance, similar to that of single-atom catalysts and better than that of nanocluster-based catalysts. We use aluminum rather than a noble metal as the principal element of the catalyst and ruthenium, which is cheaper than platinum, as the noble metal component. The design strategy provides an efficient route for the development of electrocatalysts for use in large-scale hydrogen production. Moreover, the superior hydrogen reaction evolution created by the synergistic effect of the nano-dual-phase structure is expected to guide the development of high-performance catalysts in other alloy systems.

## Teaser

A catalyst with a crystal-glass structure as a potential low-cost active catalyst during hydrogen evolution reaction is developed.

## INTRODUCTION

Electrocatalytic water splitting is a reliable technique for producing hydrogen (*1*, *2*). The lack of suitable methods for synthesizing high-performance materials (*3*, *4*) with controlled structures has limited the design of new catalytic systems (*5*). Recent studies have highlighted the good catalytic properties of amorphous materials (*6*, *7*). They have a higher density of defective sites than crystalline materials and thus have a lower energy barrier for hydrogen evolution (*8*). Moreover, amorphous materials have a robust number of active sites because of their unique electronic structure (*9*). For instance, one study found that an amorphous Au-based catalyst prepared with graphene oxide aqueous solution exhibits higher electrocatalytic activity in N_2_ reduction reactions than its crystalline counterpart (*10*). The hydrogen evolution reaction (HER) (*11*–*13*) performance of crystalline materials can be improved by tuning their lattice structure (*14*). In recent decades, crystalline multicomponent alloys have been developed (*15*, *16*). These alloys exhibit local chemical inhomogeneity (*17*), short-range ordering (*18*), and severe lattice distortion (*17*), which provide the structural basis for optimizing the catalytic reactivity in HER performance (*19*). Furthermore, excellent HER catalytic performance can be achieved by combining crystalline and amorphous phases to form nanocomposite structures; this is possible because of the unique active sites of the two phases (*20*). Research on nanoparticle or cluster structures has confirmed the advantages of small catalysts (*21*). It is reasonable to expect that, given the above advantages of high-/medium-entropy alloys and amorphous alloys in the preparation of a new class of crystal (high-/medium-entropy)–glass nano-dual-phase alloys, the synergistic effects from the two phases may contribute to excellent HER performance as dual-phase material performs in mechanical property (*22*). Therefore, integrating the crystal-glass dual-phase (*23*) structure and size effect is an optimal approach to the fabrication of new-generation catalysts.

## RESULTS

**Fig. 1.**
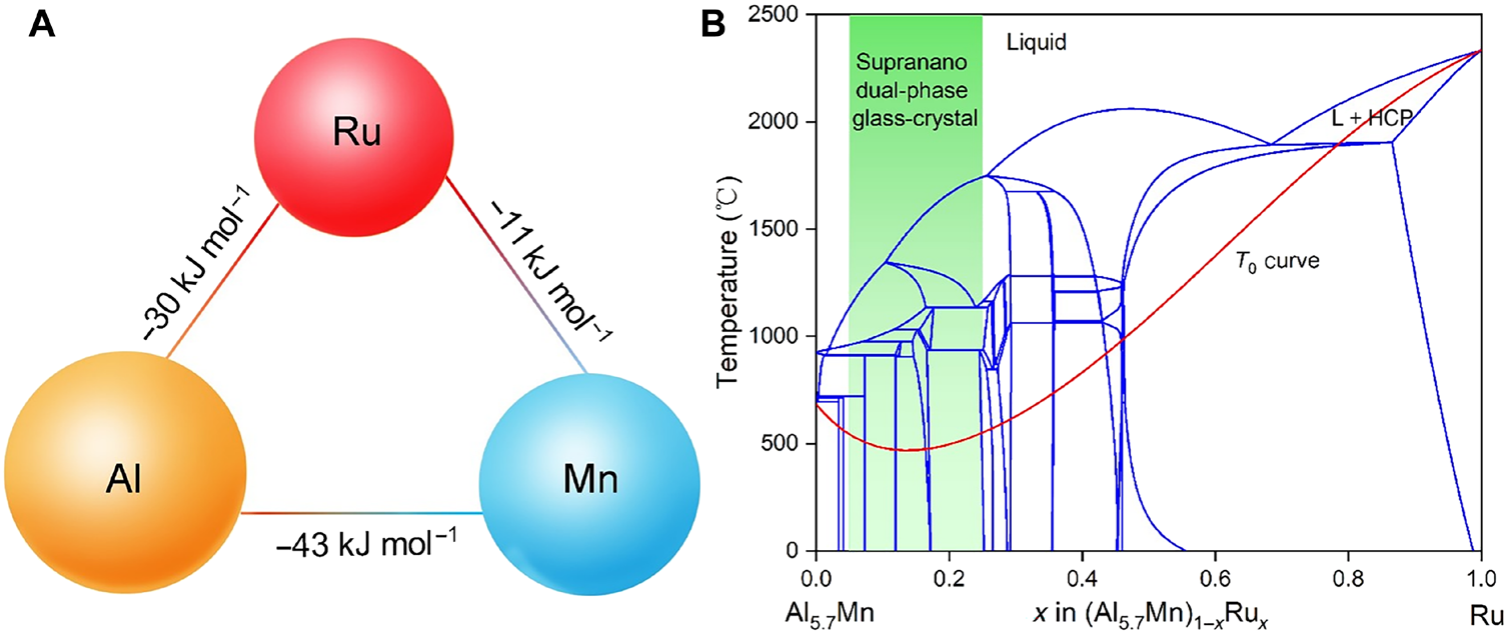
Thermodynamics-guided design of a crystal-glass nano-dual-phase Al-Mn-Ru system. (**A**) Heat of the mixing values between Al-Mn, Al-Ru, and Ru-Mn. The size of the sphere represents the relative sizes of the Al, Mn, and Ru atoms. (**B**) CALPHAD-calculated (Al_10_Mn_1_)_1−*x*_Ru*_x_* vertical section and *T*_0_ curve for the HCP (thin red line) formation of the ternary system. The calculated *T*_0_ curve reaches a minimum value at ~13 at % of Ru, revealing a weaker glass formation ability than the representative glass-forming systems. The highlighted green region corresponds to the conditions for forming a crystal-glass nano-dual-phase structure.

In this study, we design a high-performance Al-based catalyst with a crystal-glass nano-dual-phase structure. The catalyst is composed of an extremely small (~2 nm) amorphous phase and multicomponent crystalline phase. The catalyst design is based on the glass-forming ability (GFA), which we predict using established correlations between the material’s properties and empirical criteria (*4*). Among Al-based systems, Al-Ru (*24*), Al-Mn (*25*), and Ru-Mn (*24*) have large negative enthalpies of mixing (−30, −43, and −11 kJ mol^−1^, respectively). Furthermore, Al atoms are much larger than Ru and Mn atoms by 34 and 27%, respectively (Fig. 1A). Therefore, the proposed Al-Mn-Ru system has a high GFA, according to Inoue’s empirical criteria for GFA (*26*). Using the calculation of phase diagrams (CALPHAD) approach, we identify the composition region that corresponds to the crystal-glass nano-dual-phase structure. In the past few decades, CALPHAD-based thermodynamic calculations have attracted considerable attention (*27*, *28*). CALPHAD has been used for thermodynamic calculations for both equilibrium (*29*) and nonequilibrium systems, such as metastable (*27*, *28*) and amorphous phases (*30*). To simulate the crystal-glass nano-dual phase, CALPHAD can be used to design the system for a particular chemical composition range, which makes it faster and less costly than the conventional trial-and-error method. The phase formation region can be located through thermodynamic analyses; however, phase formation and transformation are usually difficult to evaluate in nonequilibrium systems (*27*, *28*). Modeling an amorphous phase is difficult and requires different levels of approximation and theoretical applications of thermodynamic driving force, kinetic barrier, classical nucleation, and amorphous phase growth (*31*). In one study, the *T*_0_ curves, which identify where the Gibbs energy of the liquid phase coincides with that of solid solutions, were calculated using the CALPHAD approach to predicting new glass-forming systems and their glass-forming regions (*30*). In Fig. 1B, the (Al_10_Mn_1_)_1−*x*_Ru*_x_* section calculated using CALPHAD is represented as a vertical line that indicates a fixed Al-to-Mn ratio. The figure also shows the calculated *T*_0_ curve for the hexagonal close-packed (HCP) phase (thin red line) of the ternary system. Unlike representative glass-forming systems, such as Ni-Ti, Ni-Zr, and Cu-Zr, whose *T*_0_ curves for terminal solid solutions plunge sharply at low temperatures (*31*), the *T*_0_ curve of the HCP structure in the Al-Mn-Ru system does not plunge; instead, it reaches its minimum value at ~13 atomic % (at %) of Ru, revealing a weak glass formation ability. In this composition region (Fig. 1B, green region), the Gibbs free energies of the liquid and crystalline phases reach maximum deviation, which can induce the formation of a nano-dual-phase structure.

**Fig. 2.**
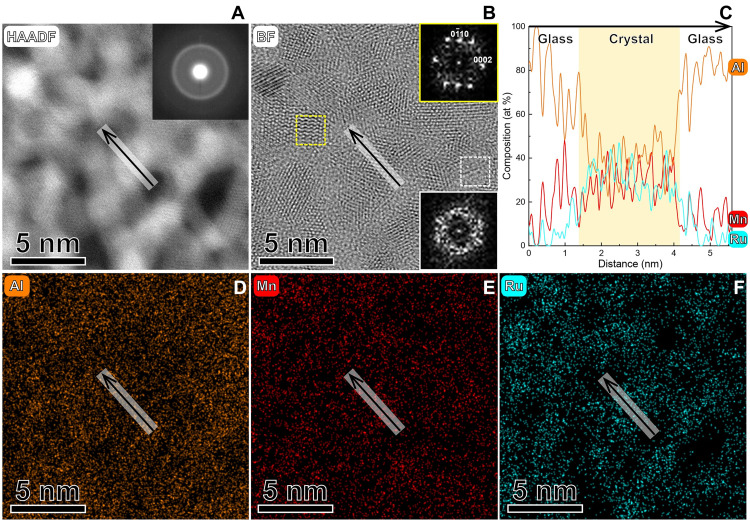
Structure and composition of the medium-entropy crystal-glass nano-dual-phase Al-based catalyst. (**A**) High-angle annular dark-field (HAADF) image probed from a cross-sectional sample. The *z*-contrast reflects the difference in atomic weight (i.e., Al-enriched amorphous regions are darker). The inset shows a typical selected-area electron diffraction pattern with a halo ring feature, attributed to the extremely small-size nanocrystals and amorphous phase. (**B**) Bright-field (BF)–STEM image probed from the same region. The fast Fourier transform image (top right inset) of the crystalline region (yellow dashed square) of an HCP pattern from the <2-1-1-0> zone axis. In contrast, the fast Fourier transform image (bottom right inset) of the white dashed square region shows a diffused pattern, indicating an amorphous structure. (**C**) 1D compositional profile, generated from (**D** to **F**) near-atomic-resolution energy-dispersive x-ray spectrometry mapping. The arrows in (A), (B), and (D) to (F) indicate the investigated region of the 1D compositional profile.

The Al-based catalyst is obtained by doping Ru into an Al-Mn system. Al-Mn-Ru catalysts are synthesized via magnetron co-sputtering of the Al_85_Mn_15_ alloy and Ru targets with 99.9 at % of purity. The investigated Al-based catalyst has a composition of Al_73_Mn_7_Ru_20_ (in atomic %), as determined by energy-dispersive x-ray spectrometry. The catalyst has a crystal-glass nano-dual-phase structure with ~2-nm-diameter amorphous regions embedded between ~2-nm-diameter globular nanograins (Fig. 2, A and B) (*32*). The detailed scanning transmission electron microscopy (STEM) characterizations are available in the Supplementary Materials. The abovementioned topology is the result of fast elemental diffusion along the triple junctions, which are occupied in the amorphous phase (*33*). The nanograins are composed of near-equiatomic Al-Mn-Ru, as shown by their one-dimensional (1D) compositional profile (Fig. 2C). The bright-field high-resolution STEM image of the nanograins reveals typical HCP planes (Fig. 2B) without the crystallographic planes of other crystalline phases. The HCP structure of the solid solution of the nanograins is related to the medium-entropy configuration of near-equiatomic composition (*18*).

Next, we discuss the glass-forming mechanism in the amorphous phase (Al_80_Mn_5_Ru_15_; in atomic %) of this nano-dual phase. Mn and Ru have large negative mixing enthalpies with Al (Fig. 1A), and thermodynamic studies (*24*, *25*) have shown that complex intermetallic phases can form in Al-Mn and Al-Rn systems with Al atomic ratios from 70 to 95%. According to one of Inoue’s empirical laws for GFA (*26*), a large negative mixing enthalpy is a critical parameter for enhanced GFA, as it promotes the generation of an amorphous structure in alloys fabricated via sputtering at high cooling rates. Therefore, the crystal-glass nano-dual-phase structure in the Al-based catalyst arises from the entropy- and enthalpy-stabilized mechanisms of the crystalline and amorphous phases, respectively.

**Fig. 3.**
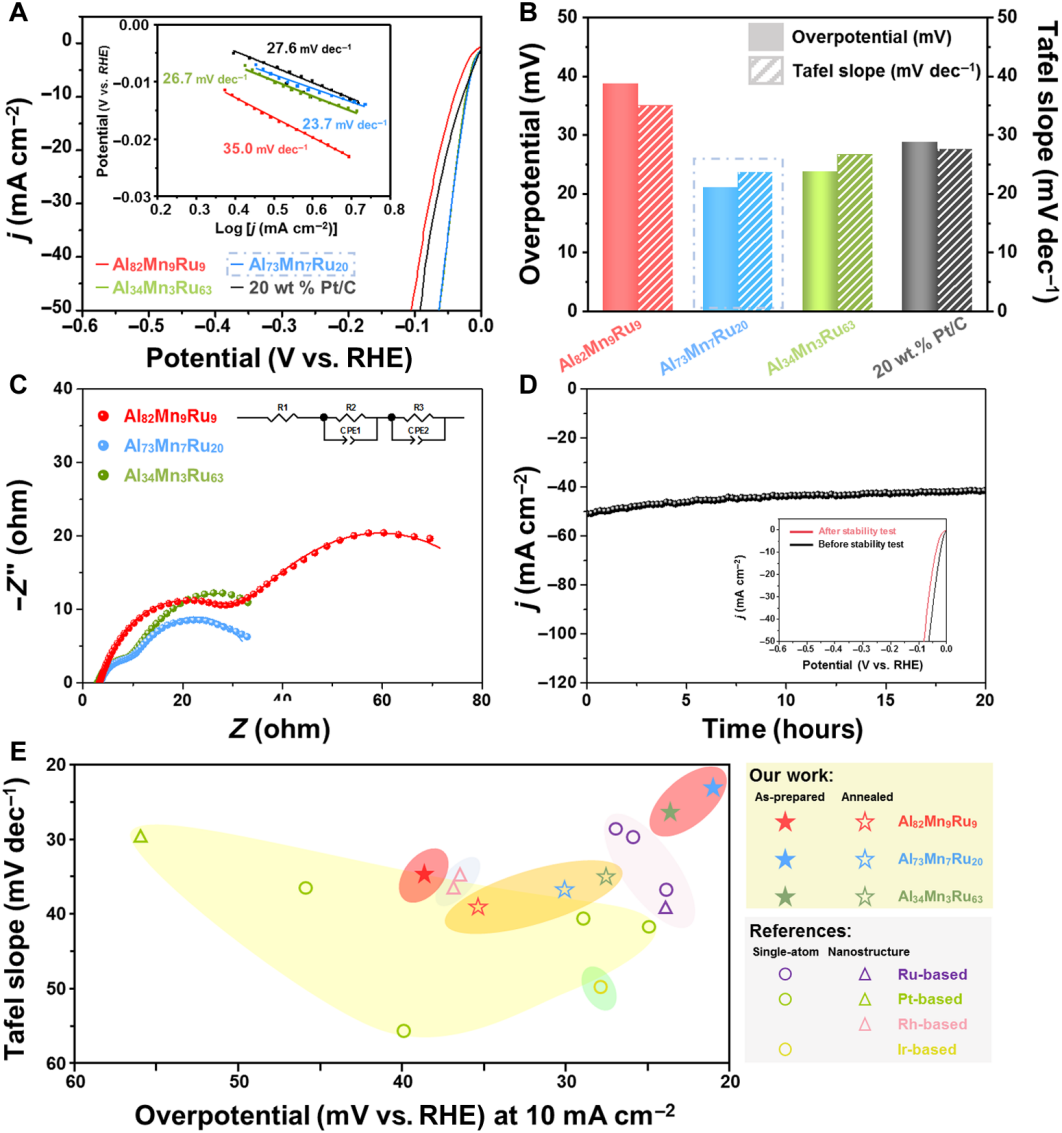
Electrocatalytic performance of the as-received samples in 1 M KOH solution. (**A**) LSV curves and Tafel slopes (inset) at a scan rate of 2 mV s^−1^ (with iR loss correction). (**B**) Overpotentials (column) and Tafel slopes (striped column) of the as-received samples and commercial Pt/C catalyst (loaded with 20 wt % of Pt) tested at 1 M KOH at a scan rate of 2 mV s^−1^ (with iR loss correction). (**C**) Nyquist plots of the as-received catalysts. The original data (dots) are modelled with a two-time constant serial model, fitted using solid lines. The top right inset is the circuit model. (**D**) Stability of the crystal-glass nano-dual-phase Al_73_Mn_7_Ru_20_ sample during HER, tested in 1 M KOH solution at 50 mA cm^−2^. The inset shows the LSV curves before and after the stability test (scanning at 2 mV s^−1^). (**E**) HER catalytic performance of the Al-Mn-Ru samples, in comparison with previously reported noble metal–based catalysts.

The nano-dual-phase catalyst supported on carbon cloth exhibits a better catalytic capacity for HER, lower overpotential, and faster kinetics than the commercial Pt/C catalyst [20 weight % (wt %) of Pt] in an alkaline solution. The linear sweep voltammetry (LSV) curves (Fig. 3, A and B) suggest that catalysts with an amorphous structure, dual-phase nanostructure, and crystalline structure that may have partial oxidation or electron transfer between heterogeneous atoms (fig. S1) (*34*–*36*) can activate HER. The overpotential at 10 mA cm^−2^ and the Tafel slope of the nano-dual-phase catalyst (21.1 mV and 23.7 mV dec^−1^ (decade^−1^), respectively) are considerably lower than those in the original amorphous samples (38.7 mV and 35.0 mV dec^−1^, respectively) and in the commercial Pt/C catalyst (28.8 mV and 27.6 mV dec^−1^, respectively). Meanwhile, the HER catalytic performance of Al_34_Mn_3_Ru_63_, which has a highly crystalline structure, is below that of the crystal-glass nano-dual-phase Al_73_Mn_7_Ru_20_.

To further examine the advantages of the nano-dual-phase structure, we anneal the samples until crystallized. All of the annealed samples, particularly the amorphous (Al_82_Mn_9_Ru_9_) and dual-phase samples (Al_73_Mn_7_Ru_20_), exhibit lower electrocatalytic performance than the nano-dual-phase catalyst (fig. S2). Thus, the superior HER catalytic performance of Al_73_Mn_7_Ru_20_ is due to its nano-dual-phase structure. Moreover, the lower catalytic performance of the highly crystalline Al_34_Mn_3_Ru_63_ indicates that higher Ru concentration weakens catalytic capacity; thus, the HER catalytic performance trends of both the original and annealed systems indicate that the crystal-glass nano-dual-phase structure has a superior capacity for water splitting, which is consistent with the results of the electrochemical impedance spectroscopy (EIS; Fig. 3C). In Fig. 3C, Nyquist plots were obtained at 0 V [versus RHE (reversible hydrogen electrode)], and the fitting curves confirm that the crystal-glass nano-dual-phase Al_73_Mn_7_Ru_20_ sample has much smaller charge transfer and mass transfer resistance than those of fully amorphous or fully crystalline samples (*37*). This characteristic enables the water molecule to accept extra energy and split into an adsorbed hydrogen atom (H_ad_) and a hydroxyl ion (OH^−^). Furthermore, the double-layer capacitance (*C*_dl_) results (fig. S3) reveal that Al_73_Mn_7_Ru_20_ exhibits a high number of active catalytic sites per unit area, which promotes HER kinetics. To reveal the catalytic ability of a single active site, the turnover frequencies (TOFs) of the dual-phase and Pt/C catalysts are calculated (fig. S4) (*38*–*47*). Given Cu^2+^ underpotential deposition (*45*, *47*), the TOF of Al_73_Mn_7_Ru_20_ is approximately 0.71∣*j*∣ and that of commercial Pt/C catalysts is approximately 0.05∣*j*∣. When current density is substituted into the above TOF calculation, Al_73_Mn_7_Ru_20_ presents a higher TOF at 50 mV (24.3 s^−1^) than Pt/C (0.98 s^−1^). The crystal-glass nano-dual-phase Al_73_Mn_7_Ru_20_ exhibits better HER catalytic performance than the noble metal-based HER catalysts (Fig. 3E) (*40*, *42*, *45*, *48*–*57*). The current-time curve at 50 mA cm^−2^ (Fig. 3D) is relatively stable, confirming the stability of the as-received Al_73_Mn_7_Ru_20_ electrocatalyst. However, if the samples are used in the acid environment, then the performance of HER becomes poorer (overpotential at 10 mA cm^−2^ > 70 mV, Tafel slope > 40 mV dec^−1^; fig. S5). This may be caused by the reduction of reactive sites (fig. S5, C to H) in acid solution.

As shown in Fig. 3D, during the long-term electrochemical test of Al_73_Mn_7_Ru_20_, there is still a weak reduction of current. To determine the origin behind the drop of current other than the peeling of film, we characterize the tested sample using energy-dispersive spectroscopy (EDS)–equipped STEM. From fig. S6, it is shown that the volume fraction of the amorphous regions is reduced after the electrochemical tests. As a consequence, the size of the medium-entropy Al-Mn-Ru nanocrystals increases from 2 to 3.8 nm (varying from 3.1 to 5.2 nm). Moreover, different from large composition difference between the amorphous and crystalline phases for the as-deposited sample, the sample after the electrochemical tests is composed of a mix of Al, Mn, and Ru in nearly equal atomic ratios. This transformation may induce a change in entropy. In the original amorphous phase, Al dominates among the three elements, but it is dissolved in an alkaline solution. As the Al dissolves, the proportions of the three elements gradually become equal, resulting in higher configurational entropy. On the basis of this inference and the Adam-Gibbs model, which describes the relationship between configurational entropy and viscosity, i.e.

$$\eta =\eta_{0}\text{exp}(\frac{A}{{\mathrm{TS}}_{\text{con}}})$$

where η is the viscosity coefficient, η_0_ is the reference viscosity coefficient under one atmosphere, *A* is a constant, *T* is the temperature, and *S*_con_ is the configurational entropy; the increase in configurational entropy will reduce the viscosity coefficient. What is more, according to the Stokes-Einstein equation, i.e.

$$D=\frac{\mathrm{kT}}{3\pi \eta a}$$

where *D* is the diffusion coefficient and *a* is the atomic distance; diffusion will be accelerated by a decrease in the viscosity coefficient. Therefore, with the dissolution of Al, atoms in the amorphous phase potentially achieve a faster migration rate, which benefits the rearrangement of the structure. The phase transition is also predicted by thermodynamics. Compared to the crystalline phase, the amorphous phase has higher energy, which makes the crystallization process an exothermic reaction (Δ*H* is negative). Coupled with the increase of configurational entropy, according to the equation of Gibbs free energy, i.e.

G=H−TS

ΔG=ΔH−TΔS (T is constant in the system)

The Gibbs free energy of the phase transition gradually becomes the most negative value at this temperature, which notably promotes the probability of the phase transition. Moreover, the remaining crystalline phase acts as a template that is able to reduce the energy requirement of the amorphous phase crystallization and accelerate the transition.

**Fig. 4.**
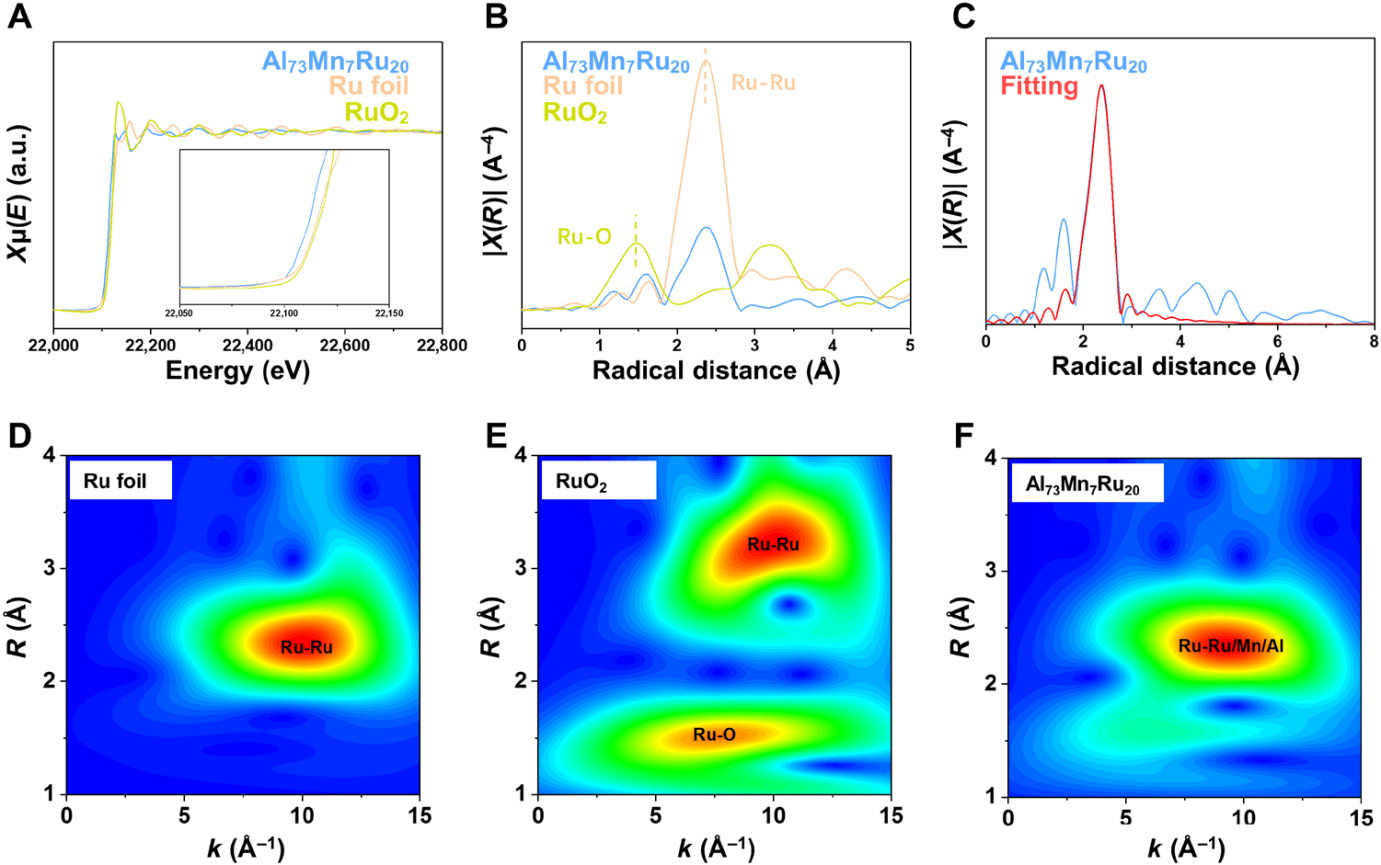
Electronic structure investigations of Al_73_Mn_7_Ru_20_. (**A**) Ru K-edge XANES of Al_73_Mn_7_Ru_20_, Ru foil, and RuO_2_; the inset figure is the absorption edge. (**B**) Fourier-transformed Ru K-edge EXAFS spectra of Al_73_Mn_7_Ru_20_ and standard samples (Ru foil and RuO_2_). (**C**) The fitting result of Fourier-transformed Ru K-edge EXAFS spectra of Al_73_Mn_7_Ru_20_. (**D** to **F**) Wavelet transform for the *k*^3^-weighted EXAFS Ru K-edge signal of Ru foil, RuO_2_, and Al_73_Mn_7_Ru_20_.

**Fig. 5.**
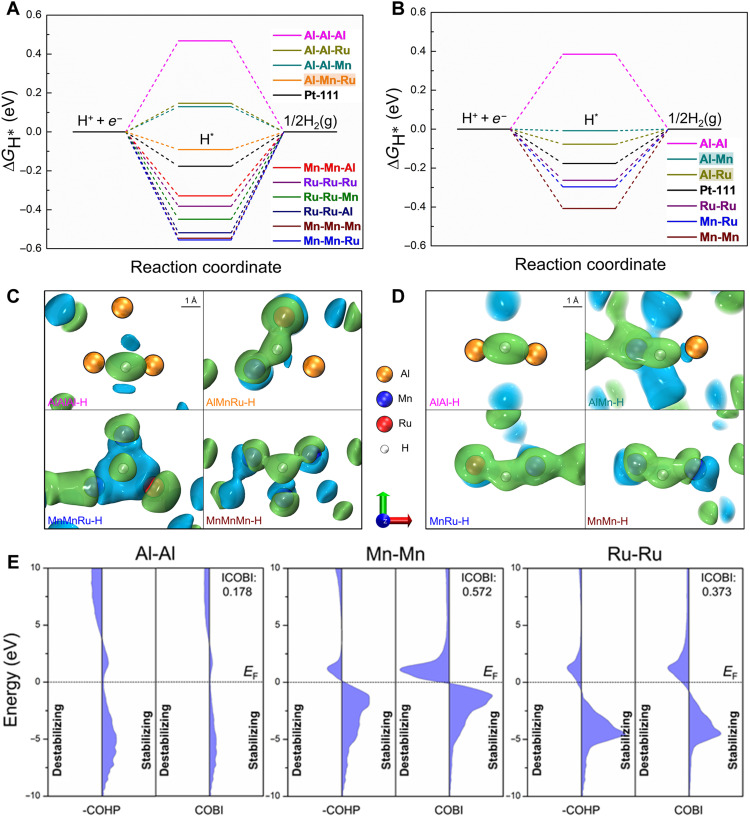
Density functional theoretical calculations for the crystalline Al-Mn-Ru phase. (**A**) Gibbs free energy (Δ*G*_H*_) profiles for different catalytic sites (hollow site with three coordinated metal atoms) at Al-Mn-Ru (001) facet. (**B**) Gibbs free energy (Δ*G*_H*_) profiles for different catalytic sites (bridge site with two coordinated metal atoms) at the Al-Mn-Ru (100) facet. Gibbs free energy (Δ*G*_H*_) for Pt (111) face-centered cubic hollow site is shown for comparison. (**C** and **D**) Charge density differences of different catalytic sites of Al-Mn-Ru (001) and (100) (isovalue = 0.00558 *e* Å^−3^ and isovalue = 0.00762 *e* Å^−3^, respectively) after H_ad_ adsorption. Except for H atom and those directly interacting with it, all other atoms in our surface reaction models were omitted for clarity. The complete adsorption configurations are shown in the Supplementary Materials (figs. S9 and S10). The cyan and lime isosurfaces represent electron depletion and accumulation, respectively, with a unit of e Å^−3^. (**E**) COHP and COBI for Al-Al, Mn-Mn, and Ru-Ru pairs, with a cutoff of 3.0 Å; the corresponding ICOBI results are shown.

The STEM characterization coupled with the discussion on both the kinetics and thermodynamics of the structural transformation explains the change in reaction activity and demonstrates the advantages of the dual-phase structure. To further elaborate the HER catalytic mechanism of the Al-Mn-Ru system, we investigated the coordination environment of Ru by the extended x-ray absorption fine structure (EXAFS) (Fig. 4). From the x-ray absorption near-edge structure (XANES) spectra of Ru in the crystal-glass nano-dual-phase catalyst (Fig. 4A), whose absorption range is affected by electron coupling, it is clear to find that Ru has lower absorption energy than Ru foil, indicating that Ru obtains more electrons from adjacent atoms. Taking the elemental content into consideration, we think that in crystal-glass nano-dual-phase structure, Al and Mn are likely to be coordinated with Ru and donate their outer electrons to Ru, which are also supported by the following fitting results. According to the fitting and wavelet transform analysis on the Ru spectra (Fig. 4, C to F), the coordination environment of Ru in the as-received catalyst is obviously close to that of Ru foil, further ruling out the probability of oxidation. In this situation, the fitting result in table S1 shows the varying coordination number of Ru-Ru, which is notably reduced from 12 to 3.7 ± 0.3, while, in turn, Ru-Al and Ru-Mn increase to 5.5 ± 0.5 and 4.0 ± 0.2, respectively. On this occasion, Ru-Ru is proven to be replaced by Al-Ru and Mn-Ru (Al-Mn-Ru), whose advantage was further found by following density functional theoretical calculations. We select two exposed surfaces of Al-Mn-Ru, namely, the (001) and (100) facets, to study the Volmer-Heyrovsky reaction (*58*) and investigate Pt (111) for comparison. For the Volmer step of H_2_O adsorption and dissociation, the adsorption energy (*E*_water_) of an H_2_O molecule adsorbed on top of a single-metal atom (Al, Mn, and Ru) on each exposed surface is shown in fig. S7. All of the adsorption sites except the Al sites on the Al-Mn-Ru (001) facet exhibit better *E*_water_ than Pt (111), indicating that the water adsorption process is most effective on the exposed surfaces, substantially enhancing catalytic efficiency. The atomic configuration and corresponding charge density difference on the Al-Mn-Ru (001) and (100) facets after H_2_O adsorption at different sites are shown in fig. S8. Given the linear Brønsted-Evans-Polanyi relationship between *E*_water_ and the dissociative kinetic barrier of H_2_O, *E*_water_ is used as an activity descriptor for the H_2_O dissociation kinetic barrier. For example, the lowest *E*_water_ (−0.63 eV) corresponding to the Mn site on the Al-Mn-Ru (100) facet indicates the smallest kinetic barrier to H_2_O dissociation. The Gibbs free energies (Δ*G*_H*_) of H adsorbed on different catalytic sites during the Heyrovsky adsorption and desorption step are presented in Fig. 5 (A and B). For the Al-Mn-Ru (001) facet, 10 possible combinations of hollow catalytic sites are considered. For the Al-Mn-Ru (100) facet, six possible combinations of bridge catalytic sites are considered. The corresponding local chemical environment of different H* adsorption sites at the (001) facet and the (100) facet of the crystalline Al-Mn-Ru phase is shown in figs. S9 and S10, respectively. The results suggest that Al has substantially weaker proton affinity than Mn and Ru, that is, the Al-free catalytic sites exhibit energetically stable H_ad_ states [Δ*G*_H*_(Mn-Mn-Mn) = −0.546 eV]. The catalytic sites with at least two Al atoms present positive Δ*G*_H*_ values, which means that they are not favorable for H adsorption [Δ*G*_H*_(Al-Al) = 0.385 eV]. The catalytic sites with only one Al atom may exhibit ideal H adsorption and desorption, as some of them have nearly zero Δ*G*_H*_ [e.g., Δ*G*_H*_(Al-Mn) = −0.008 eV]. A comparison of the electron density differences under the same isovalues (Fig. 5, C and D, and fig. S11) reveals that the bonding strength between an H atom and metal atoms in the catalytic site is consistent with the Gibbs free energy results. To further clarify the bonding nature of Al-Mn-Ru, we perform quantum chemistry bonding analyses via the crystal orbital Hamilton population (COHP) (*59*) and crystal orbital bond index (COBI) (*60*) methods (Fig. 5E and fig. S12). We quantitatively study the bond orders of the atomic pairs in Al-Mn-Ru via the integrated COBI (ICOBI) method. The ICOBI results suggest that the covalent bonding of the Al-Al pair is relatively weak (ICOBI value: 0.178), whereas the Mn-Mn and Ru-Ru pairs exhibit strong covalent bonding (0.572 and 0.373, respectively), indicating that the Al-Mn-Ru has some intermetallic bonding states. Overall, in the medium-entropy alloy component of the nano-dual-phase structure, the near-equiatomic property of Al_73_Mn_7_Ru_20_ is favorable to the formation of highly efficient hydrogen evolution bonds, such as Al-Mn-Ru, Al-Mn, and Al-Ru bonds, which have suitable H_2_O adsorption ability and fast H_2_ release rate. Here, we simulated the crystalline phase (Al_33.3_Mn_33.3_Ru_33.3_; in atomic %) in the crystal-glass nano-dual-phase Al-Mn-Ru alloy, which had already exhibited ultrahigh HER performance at specific adsorption sites (1 ≤ number of Al atom < 2). Furthermore, more active sites may exist because of the synergistic effect of crystal-glass dual-phase structure (*20*). In homogeneous structures, such as amorphous or crystalline structures, induced by fluctuations in elemental concentrations, inert or overly adsorbed bonding states become dominant, restricting the Volmer-Heyrovsky process. Furthermore, because of the extremely small size of the dual-phase structure, the synergy (*61*) between the crystal and glass phases in the Al_73_Mn_7_Ru_20_ can occur easily, further enhancing catalytic performance. In contrast, the single-phase amorphous and highly crystalline samples exhibit a substantially weaker synergistic effect (Fig. 3). Therefore, the crystal-glass nano-dual-phase structure endows the Al_73_Mn_7_Ru_20_ with superior catalytic activity for electrochemical HER.

## DISCUSSION

In summary, we develop an Al-based catalyst (Al_73_Mn_7_Ru_20_) with a crystal-glass nano-dual-phase structure as a potential low-cost active catalyst to replace noble metal–based HER catalysts. The nanostructure is composed of ~2-nm amorphous regions embedded between ~2-nm Al-Mn-Ru crystals. The Al-based catalyst outperforms most commercial catalysts and shows excellent HER catalytic performance, with an overpotential of 21.1 mV at a current density of 10 mA cm^−2^ and a Tafel slope of 23.7 mV dec^−1^. This performance can be explained by the synergistic effect of the amorphous and medium-entropy crystalline phases and the ultrahigh density of the active sites that is the result of its extremely small size and the composition of the phases. We present an efficient thermodynamics-based approach to guiding the material design of a crystal-glass nano-dual-phase structure with outstanding electrochemical properties. The nano-dual-phase electrocatalyst mechanism can also be applied to other catalytic systems. The concept of an extremely small crystal-glass nanostructure will facilitate the development of new-generation catalysts with convenient and scalable preparation techniques.

## MATERIALS AND METHODS

### Fabrication of the Al-based catalyst

The AlMnRu catalyst was prepared using one magnetron co-sputtering. We selected Al_85_Mn_15_ (in atomic %) alloy and Ru with a purity of 99.9% as the sputtering targets. An Al_73_Mn_7_Ru_20_ (in atomic %) catalyst with a thickness of 0.9 μm was then deposited on Si (001) and carbon cloth substrates with the same size using magnetron co-sputtering. During this process, the argon pressure was 0.2 Pa, the deposition rate was 0.25 nm s^−1^, the substrate bias voltage was −60 V, and the substrate temperature was maintained at 100°C.

### Structural and compositional characterization

The TEM image and the corresponding selected-area electron diffraction patterns confirmed the dual-phase microstructure. The microstructure and composition of the Al-based catalysts were investigated using STEM. The final milling voltage/current was 2 kV/23 pA, which was sufficiently small to reduce the focused ion beam damage. We used a probe aberration–corrected FEI Titan Themis, operated at 300 kV, for the high-resolution STEM imaging and EDS analyses. For the high-angle annular dark-field (HAADF) imaging, a probe semiconvergence angle of 23.8 mrad and inner and outer semicollection angles ranging from 73 to 200 mrad were used. For the EDS mapping, the dwell time was 10 μs per pixel with a map size of 1024 pixels by 1024 pixels; it took 100 frames (21 min) of EDS mapping to reach an appropriately high signal-to-noise ratio.

X-ray photoelectron spectroscopy, conducted with a Thermo Fisher Scientific K-Alpha^+^ spectrometer operating at 100 W with a monochromatic Al Kα x-ray (1486.6 eV), was used to investigate the surface electronic states and transformations during the electrocatalytic process. A high vacuum (*P* < 10^−8^ mbar) with 50-eV pass energy was prepared for analyzing the samples. The obtained x-ray photoelectron spectroscopy was calibrated with C 1s peak whose binding energy was located at 284.8 eV. Ru K-edge analysis was conducted with Si (311) crystal monochromators at the BL14W1 beamlines at the Shanghai Synchrotron Radiation Facility (Shanghai, China). Before the analysis at the beamline, samples were pressed into thin sheets with 1 cm in diameter and sealed using a Kapton tape film. The XAFS spectra were recorded at room temperature using a four-channel silicon drift detector Bruker 5040. Because of the property of sample, Ru K-edge EXAFS spectra were recorded in fluorescence mode. Negligible changes in the line shape and peak position of Ru K-edge XANES spectra were observed between two scans taken for a specific sample. The EXAFS spectra of these standard samples (foil and corresponding oxides) were recorded in transmission mode. The spectra were processed and analyzed by the software codes Athena and Artemis (*62*).

### Electrochemical measurement

Throughout the measurement process, the electrochemical catalytic performance was conducted in 1 M KOH solution through an electrochemical workstation (CHI 660E) with three electrodes; the workstation consisted of a carbon cloth with different nanostructures as the working electrode, a graphite rod (*d* = 6 mm) as the countering electrode, and a saturated calomel electrode as the reference. All of the potential was transferred to a reversible hydrogen electrode based on the Nernst equation with a correction of *iR* (*i* is current, *R* is the resistance of solution) loss, which was obtained through the electrochemical station and compensated for by calculation. The pH (14.09 to 14.12) was tested through a corrected Sartorius pH meter (PB-21). In addition, the saturated calomel electrode was calibrated in H_2_-saturated 1 M KOH solution (fig. S13) with a scanning rate of 2 mV s^−1^ to further guarantee the accuracy of the potentials. For comparison, a 5-mg commercial Pt/C catalyst (loaded with 20 wt % of Pt; Sigma-Aldrich) was dispersed in a solution consisting of 0.5-ml ethanol [ACS (American Chemical Society) grade; Anaqua] and 10-μl Nafion (5 wt %; Sigma-Aldrich) under 30 min of ultrasonication. Then, 10 μl of the as-dispersed solution was loaded and dried on the same carbon cloth used for other samples at room temperature. After 10 cycles of cyclic voltammetry with a scanning rate of 100 mV s^−1^, the reductive sweep was measured using LSV with a scanning rate of 2 mV s^−1^. The linear section of the Tafel slope was calculated using the following equation: η = *a* + *b* log [*j*] (where η is the overpotential obtained through the LSV, *a* is the Tafel constant, *b* is the Tafel slope, and log[*j*] is the logarithm of the current density, which was calculated according to geometric surface area) (*63*). EIS was measured less than around 0 V (versus RHE) from 0.01 to 10^5^ Hz in 1 M KOH solution. The obtained Nyquist plots were fitted by the two-time constant serial model. The experimental protocol is similar to that in other reports (*64*). Series cyclic voltammetry with different scanning rates (20, 40, 60, 80, 100, and 120 mV s^−1^) was used to roughly evaluate the *C*_dl_. Then, on the basis of the capacitance value and the geometric area, the electrochemical active surface area (ECSA) was obtained using the following equation: $\text{ECSA}=\frac{C_{\text{dl}}}{C_{s}}*s$ [where *C*_s_ is 40 μF cm^−2^ and *S* is geometric surface area (*65*)]. The HER stability was tested under a current density of 10 mA cm^−2^ without *iR* loss.

### Density functional theory calculation

Density functional theory calculations were conducted using the Vienna Ab Initio Simulation Package (VASP) (*66*). The projector augmented wave pseudo-potentials (*67*) and Perdew-Burke-Ernzerhof functionals (*68*) were the inputs for the VASP. The energy cutoff of the plane wave was set at 500 eV. Initially, a unit cell with a metallic Ru HCP structure (*a* = *b* = 2.705 Å, *c* = 4.281 Å, *a* = *b* = 90°, and *g* = 120°) was selected on the basis of the experimental results. A 4 × 4 × 2 supercell containing 96 atoms was built; then, the Ru atoms were randomly substituted by Al and Mn atoms. The resultant formula was Al_32_Mn_32_Ru_32_ (1:1:1). A Monkhorst-Pack grid with a size of 4 × 4 × 3 was used to sample the Brillouin zone when optimizing the lattice parameters (*69*). The optimized geometry was obtained by minimizing the forces on the atoms to less than 0.015 eV Å^−1^ (fig. S14). The surface models of AlMnRu were built with 15 Å of vacuum space, and the sizes of the Monkhorst-Pack grid used for sampling the Brillouin zone were 4 × 4 × 1 and 3 × 3 × 1 for the AlMnRu (001) and AlMnRu (100), respectively. A self-consistent field convergence with a threshold value of 10^−7^ eV was used for all of the calculations. The quantum chemistry bonding analyses (Fig. 5E and fig. S12) were performed using the schemes of the COHP (*59*) and COBI (*60*) methods, which were implemented in the Local Orbital Basis Suite Towards Electronic-Structure Reconstruction (LOBSTER) code (*70*).

The water adsorption energies (*E*_water_) were calculated using the following equation

$$E_{\text{water}}=E_{\text{surface}+H_{2}O}-E_{\text{surface}}-E_{H_{2}O}$$

where the *E*_surface_ and the *E*_surface+H_2_O_ are the total energies of the surface before and after water adsorption and *E*_H_2_O_ is the energy of a free water molecule. The Gibbs free energies of hydrogen adsorption (Δ*G*_H*_) were calculated using the following equation

$$∆G_{H^{*}}=∆E_{H^{*}}+∆E_{\text{ZPT}}-T∆S_{H}$$

where ∆*E*_H*_, ∆*E*_ZPT_, *T*, and ∆*S*_H_ represent the adsorption energy, zero-point energy change, temperature, and entropy differences of H between the H_ad_ state (H*) and gas phase hydrogen (H_2_), respectively. ∆*E*_ZPT_ and ∆*S*_H_ were obtained in the previous study (*71*), and ∆*G*_H*_ was calculated using the following equation

$$∆G_{H^{*}}=∆E_{H^{*}}+0.24\;\text{eV}$$

Charge density differences were obtained using the following equation

$$∆\rho =\rho_{\text{AB}}-\rho_{A}-\rho_{B}$$

where ρ_AB_ is the charge density of the total sorbate and substrate system and ρ_A_ and ρ_B_ are the charge densities of the sorbate and substrate, respectively. A VASPKIT code was used to postprocess the data (*72*).

### CALPHAD approach (systems, *T*_0_)

Our Al-Mn-Ru thermodynamic database was based on the well-optimized Al-Mn (*73*) and Al-Ru (*74*) phase diagrams. As the experimental phase diagram was incomplete, the Mn-Ru system was described by extrapolating the unary parameters (*75*, *76*). Detailed thermodynamic models for the liquid, solution, intermetallic, and ordered phases have been developed in the literature (*77*, *78*) but are not listed here. Modeling the amorphous phase, which is necessary for the exploration of new glass formation materials, requires great effort and special care. Researchers have used different levels of approximation to consider the thermodynamic driving force, kinetic barrier, classical nucleation, and growth theory of amorphous phases (*31*). In this study, we adopted Chen’s interpretation of *T*_0_ curves to predict new glass-forming systems and their glass-forming regions (*30*).
