# Supplementary material for: A crystal glass–nanostructured Al-based electrocatalyst for hydrogen evolution reaction
Source: Sci Adv. 2022 Nov 4;8(44):eadd6421. doi: 10.1126/sciadv.add6421 (PMC9635819; doi:10.1126/sciadv.add6421)
Supplement: Supplementary file 1 — Supplementary Text Figs. S1 to S14 Table S1 [file sciadv.add6421_sm.pdf]

Science Advances
AAAS

Supplementary Materials for

A crystal glass–nanostructured Al-based electrocatalyst for hydrogen evolution reaction

Sida Liu *et al.*

Corresponding author: Ge Wu, gewuxjtu@xjtu.edu.cn; Yangyang Li, yangli@cityu.edu.hk; Jian Lu, jianlu@cityu.edu.hk

Sci. Adv. **8**, eadd6421 (2022)
DOI: 10.1126/sciadv.add6421

This PDF file includes:

Supplementary Text
Figs. S1 to S14
Table S1

Supplementary Text

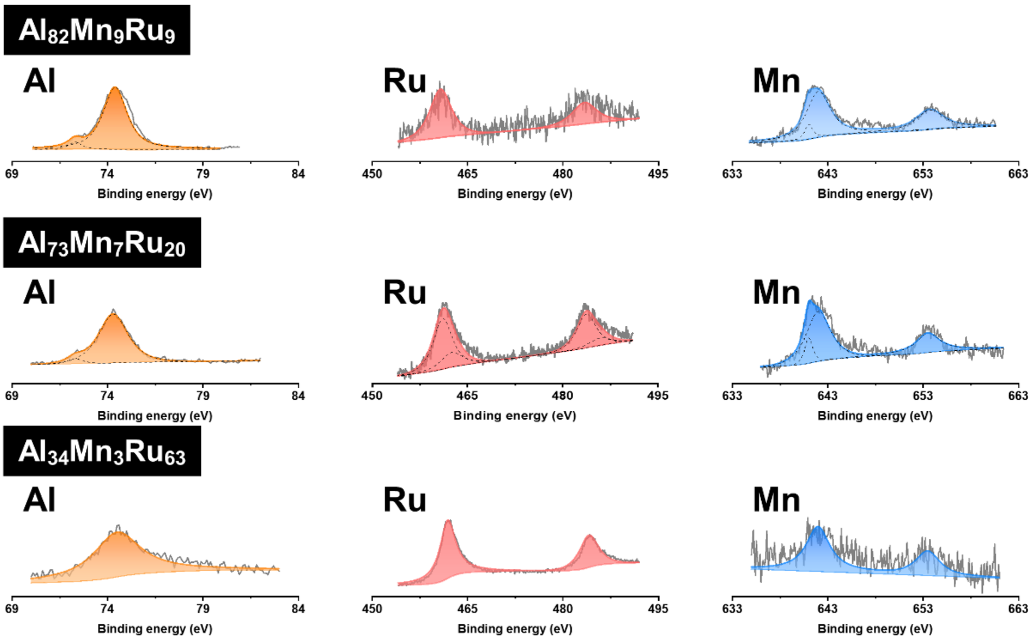

Figure S1. X-ray photoelectron spectra of the electrocatalysts.

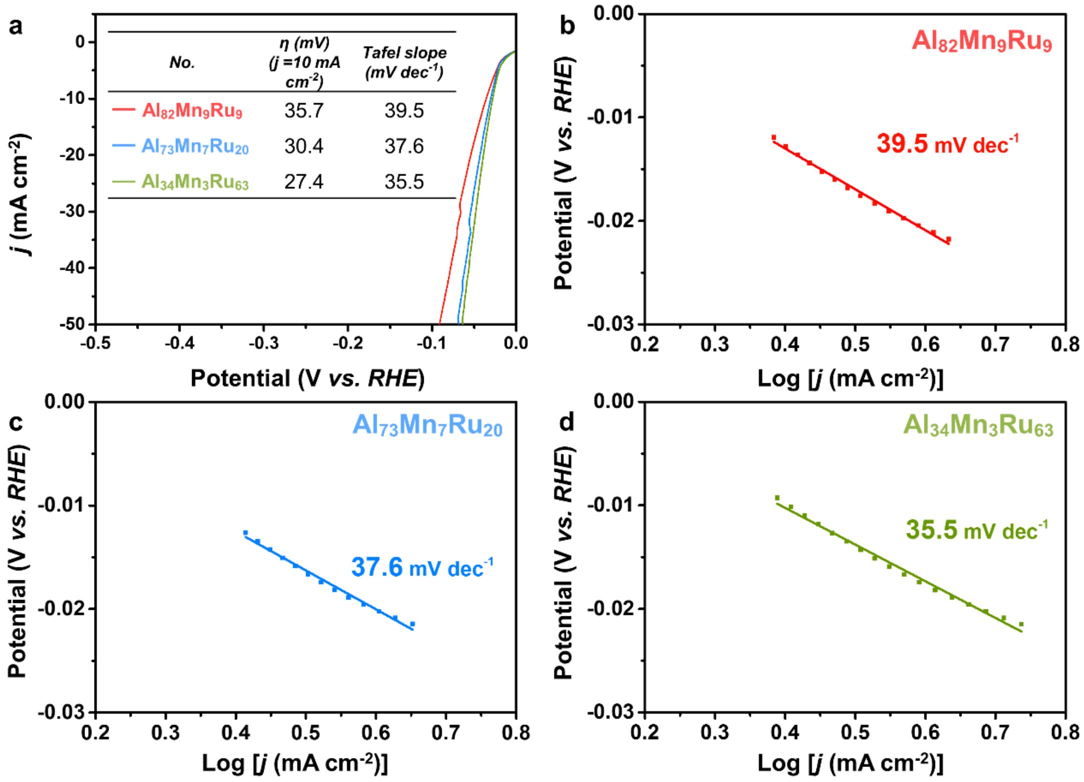

Figure S2. Electrocatalytic performance of the annealed samples in 1M KOH solution. a. Linear sweep voltammetry and Tafel slopes at a scanning rate of 2 mV s^{-1} using the iR correction method described in the *Electrochemical Measurement* section. **b-d.** The Tafel slopes of the annealed samples of the three catalysts.

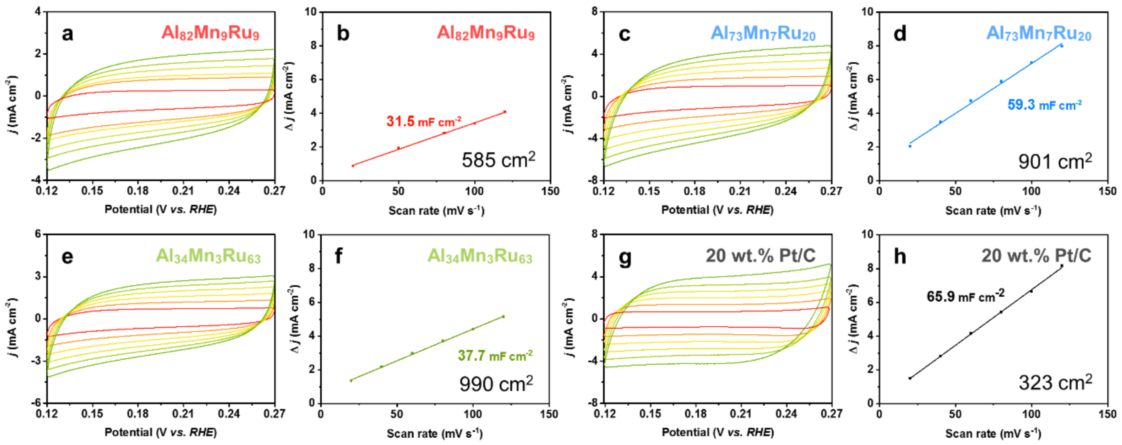

Figure S3. Electrochemical active surface area of as-received catalyst and commercial 20 wt.% Pt/C. a, c, e, and **g**: The cyclic voltammetry of the three as-received samples and the commercial Pt/C catalyst at different scanning rates (20 to 120 mV s^{-1} with 20 mV s^{-1} intervals) among 0.12-0.27 V (*vs. RHE*). **b, d, f**, and **h,** the determination of double layer capacitance, were calculated based on the cyclic voltammetry data (**a, c, e,** and **g**) at *E* = 0.195 V (*vs. RHE*) of the three as-received samples and commercial Pt/C catalyst.

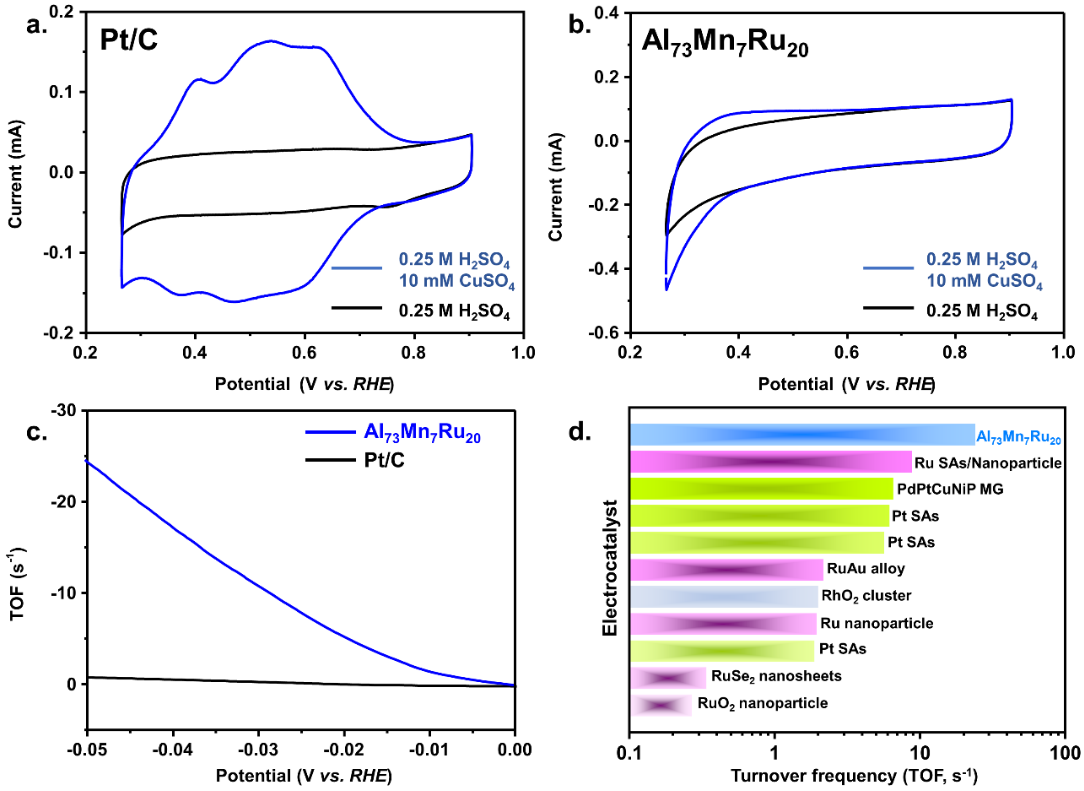

Figure S4. Turnover frequencies measured through cycling voltammetry of different solutions at a scanning rate of 10 mV s^{-1}. a. Commercial 20 wt.% Pt/C; **b**. $Al_{73}Mn_7Ru_{20}$; **c**. TOF curve calculated based on current density; and **d**. TOF value comparison among different noble alloy catalyst.

The turnover frequency of $Al_{73.3}Mn_{6.4}Ru_{20.3}$ and Pt/C was estimated using the following equation:

$$TOF = \frac{i}{2Fn} = \frac{|j|A}{2Fn}$$

where i is the current (mA) from the polarization curve (LSV) in Fig. 1, F is the Faraday constant (96485 C mol^{-1}), n (mmol) is the molar value of the active sites, $|j|$ is the absolute value of current density (mA cm^{-2}), A is the geometric area (cm^2), and the coefficient 1/2 reflects the fact that one hydrogen molecule needs two electrons.

The molar value of active sites was obtained through the deposition of Cu^{2+}/Cu. As shown in Fig. S4, the anodic peak around 0.38 V (*RHE*) was ascribed to the Cu stripping peaks. The deposition of copper changes the area of the cycling voltammetry. Based on this difference, the quantity of active sites was roughly estimated using the following formula:

$$n = \frac{Q_{Cu}}{2F}$$

where Q_{Cu} is the difference in the area of cycling voltammetry and F is the Faraday constant (96485 C mol^{-1}). The number of active sites in $Al_{73.3}Mn_{6.4}Ru_{20.3}$ was 0.85 mC, whereas the value in commercial 20 wt.% Pt/C catalyst was 3.9 mC. Therefore, the TOF value was

$$For\, Al_{73.3}Mn_{6.4}Ru_{20.3}, \qquad TOF = \frac{|j|A}{2Fn} = \frac{|j| * 0.6075\, cm^2}{0.85\, mC} = 0.71|j|$$

$$For\ Pt-C, \qquad TOF = \frac{|j|A}{2Fn} = \frac{|j| * 0.196\, cm^2}{3.9\, mC} = 0.05|j|$$

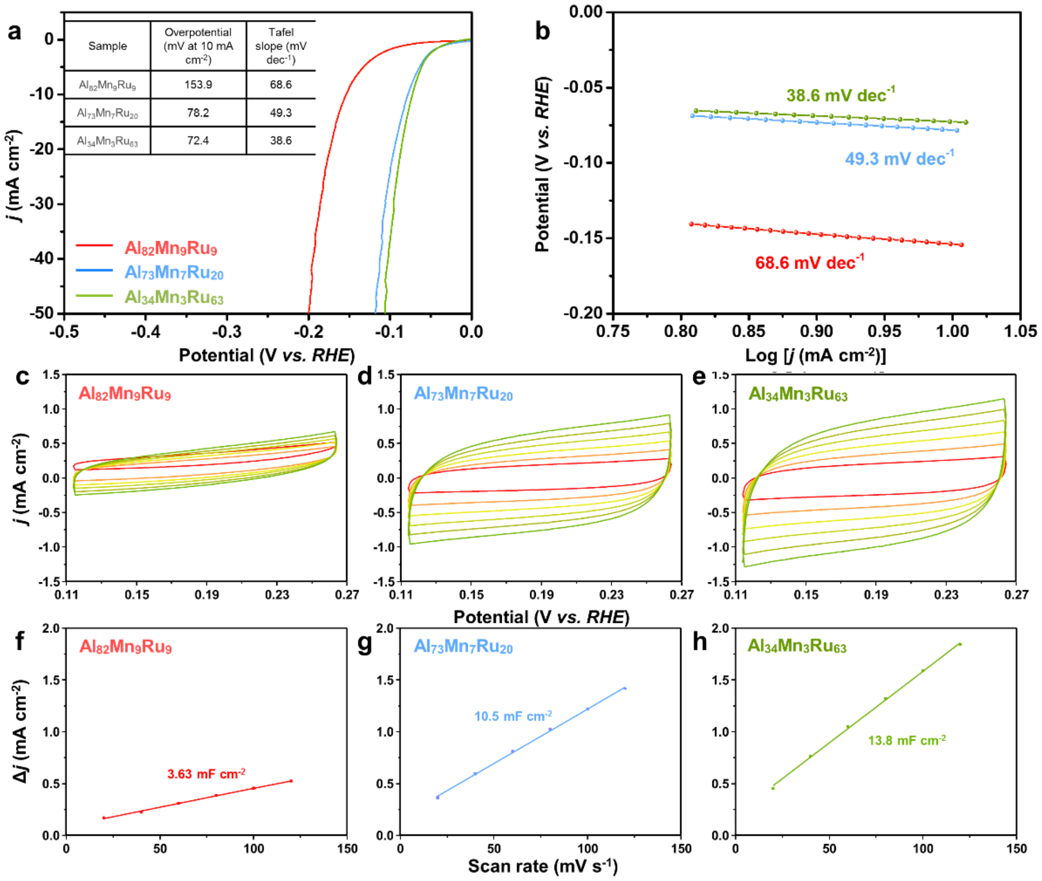

Fig. S5. Electrocatalytic performance of the as-received samples in 0.5M H_2SO_4 solution. a. LSV curves at a scan rate of 2 mV s^{-1} (with iR loss correction), the inset table shows the overpotential (10 mA cm^{-2}) and Tafel slope. b. Tafel slope of samples tested in 0.5M H_2SO_4. c, d, and e: The cyclic voltammetry of the three as-received samples at different scanning rates (20 to 120 mV s^{-1} with 20 mV s^{-1} intervals) among 0.11-0.27 V (*vs. RHE*). f, g, and h: The determination of double layer capacitance, which were calculated based on the cyclic voltammetry data (c, d, and e) at E = 0.189 V (*vs. RHE*) of the three as-received samples.

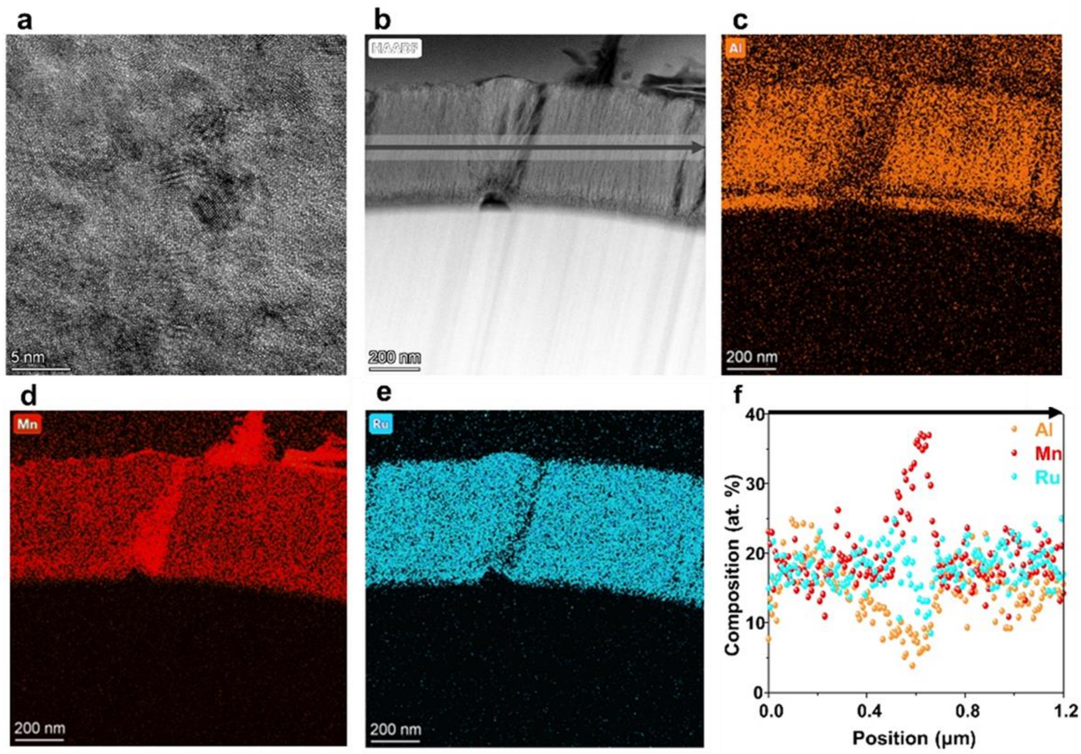

Fig S6. Structure and composition of $Al_{73}Mn_7Ru_{20}$ after the test in 1 M KOH. a. BF-STEM image probed from a cross section. b. High-angle annular dark-field (HAADF) image probed from the same region. c-e. Energy-dispersive X-ray spectrometry mapping. f. 1D compositional profile, generated from (c-e). The arrows in b indicate the region of the 1D compositional profile investigated in this analysis.

Table S1. Fitting result of Fourier-transformed Ru K-edge EXAFS spectra of $Al_{73}Mn_7Ru_{20}$ and Ru foil. Ru K-edge EXAFS. CN is the average coordination number, ΔR is the difference between fitted R and theoretical bond length, R is the distance from absorber atom, and σ^2 the Debye–Waller factor. R-factor denotes a quality factor of the fitting, and ΔE_0 the energy shift from the absorption edge energy E_0.

Sample	Path	CN	R, Å	D-W factor (σ^2, Å^2)	ΔE_0, eV	R-factor
$Al_{73}Mn_7Ru_{20}$	Ru-Ru	3.7 ± 0.3	2.72 ± 0.01	0.006 ± 0.001	2.7 ± 0.7	1.1%
	Ru-Mn	4.0 ± 0.2	2.60 ± 0.02	0.005 ± 0.001	8.3 ± 0.8	
	Ru-Al	5.5 ± 0.5	2.65 ± 0.02	0.006 ± 0.003	8.5 ± 1.6	
Ru foil	Ru-Ru	12	2.71 ± 0.01	0.004 ± 0.001	-4.8 ± 0.9	1.8%

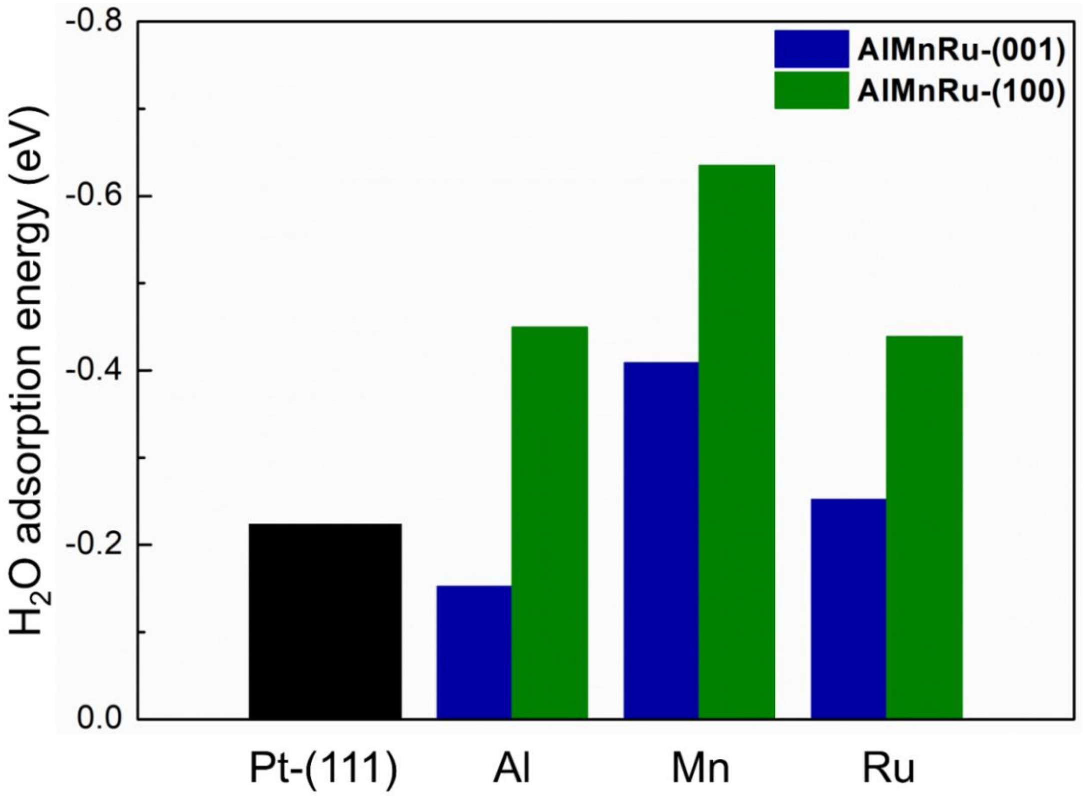

Fig. S7. DFT calculated adsorption energies of H_2O molecules adsorbed on Pt-(111) and different sites on AlMnRu (001) and AlMnRu (100).

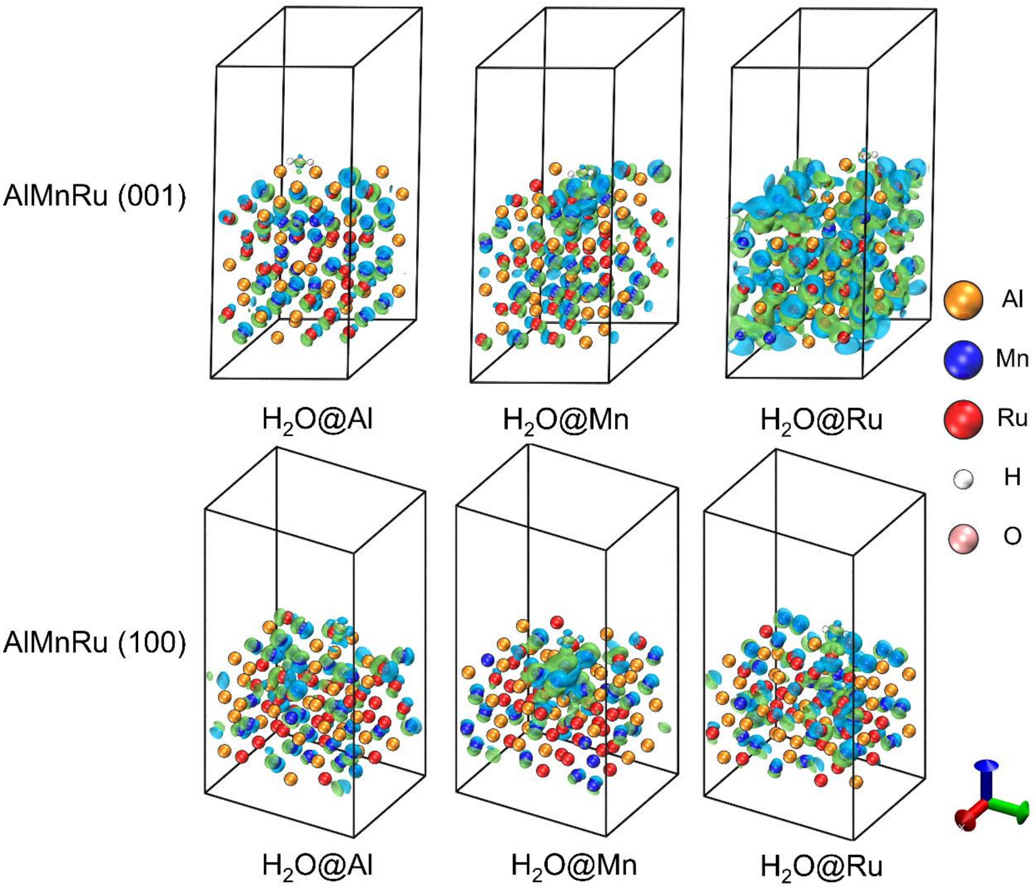

Fig. S8. Atomic configurations and the corresponding charge density differences on AlMnRu (001) and (100) facets after H_2O adsorption at different sites. The cyan and lime isosurfaces represent the depletion and accumulation of electrons in units of e $Å^{-3}$, respectively.

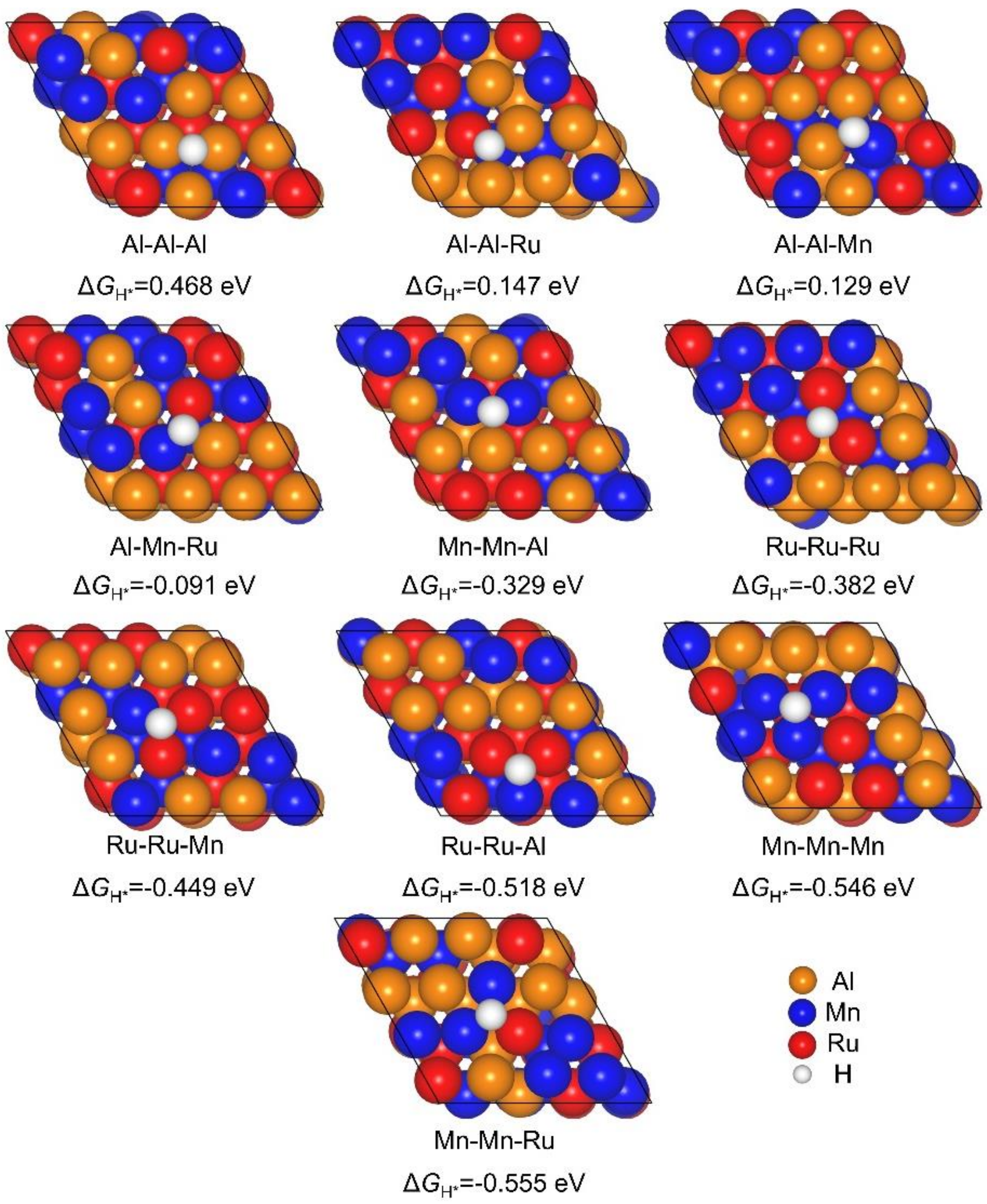

Fig. S9. Local chemical environment of different H* adsorption sites at the (001) facet of the crystalline Al-Mn-Ru phase. ΔG_{H*} is the corresponding Gibbs free energy of H.

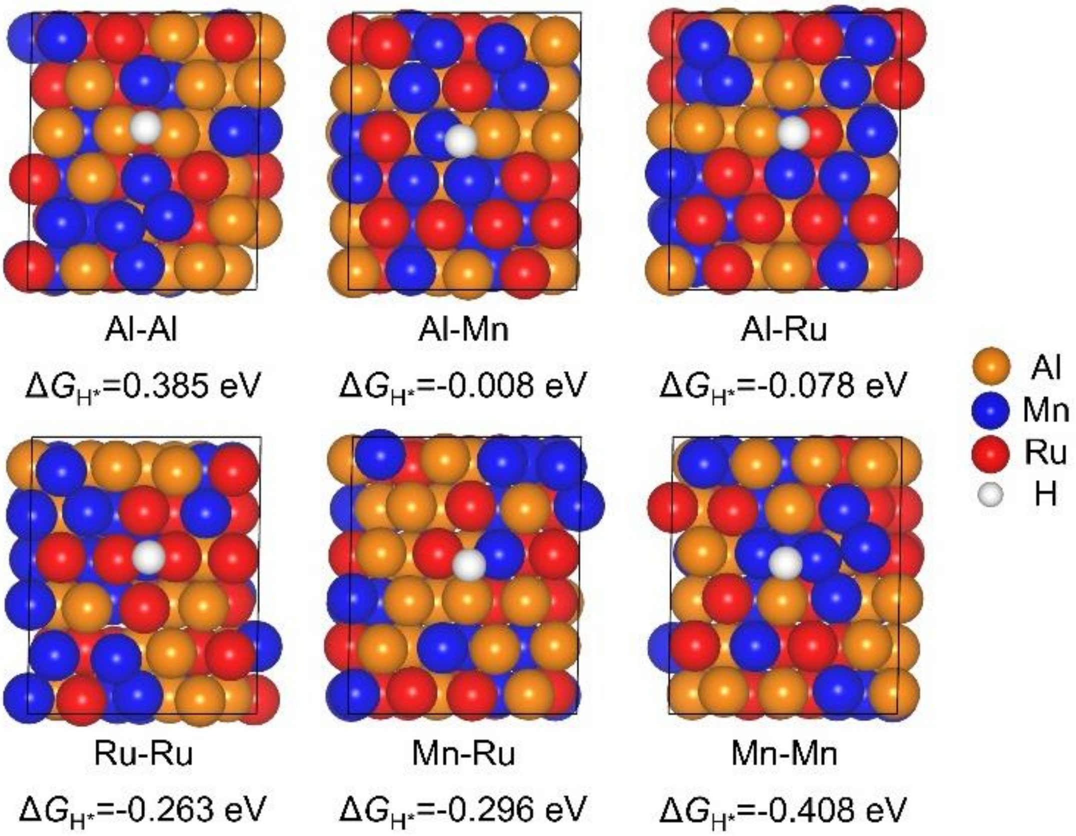

Fig. S10. Local chemical environment of different H* adsorption sites at the (100) facet of the crystalline Al-Mn-Ru phase.

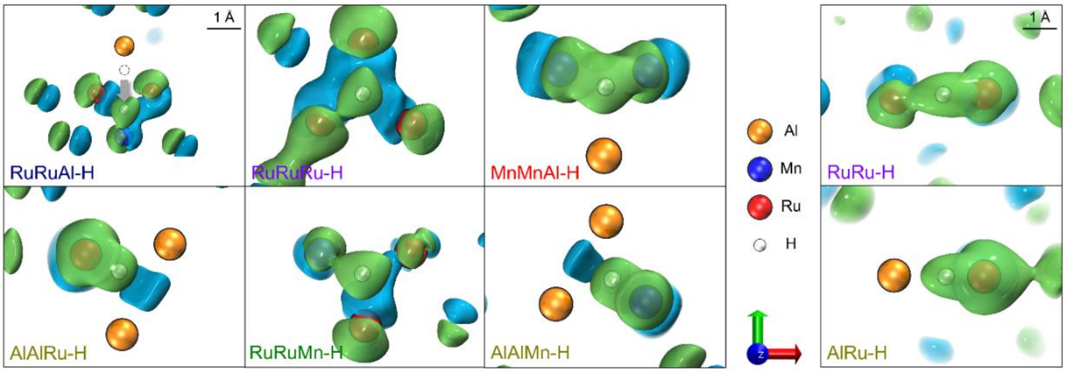

Fig. S11. Charge density differences of different catalytic sites after H_{ad} adsorption. The cyan and lime isosurfaces represent the depletion and accumulation of electrons with the unit of e $Å^{-3}$, respectively. a. AlMnRu (001), isovalue = 0.00558 e $Å^{-3}$. b. AlMnRu (100), isovalue = 0.00762 e $Å^{-3}$. Note that in catalytic site Ru-Ru-Al, H_{ad} deviated from its initio position (gray dashed circle) to an adjacent catalytic site Ru-Ru-Mn. The occurred because the proton affinity of Al is much weaker than that of Mn or Ru, i.e., H adsorbed on catalytic site Ru-Ru-Mn possesses a lower energy than H adsorbed in catalytic site Ru-Ru-Al.

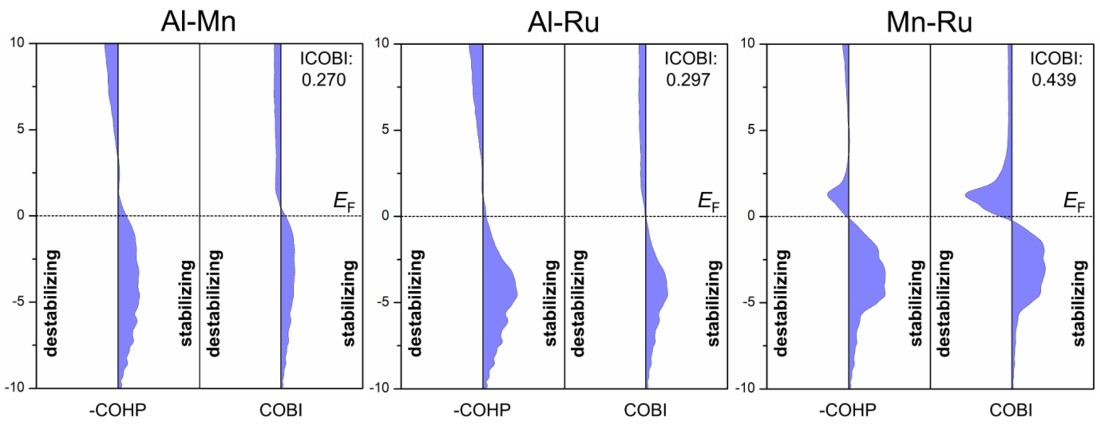

Fig. S12. Crystal orbital Hamilton population (COHP) and crystal orbital bond index (COBI) for Al-Mn, Al-Ru and Mn-Ru pairs with a cutoff of 3.0 Å. The corresponding integrated COBI (ICOBI) are also shown.

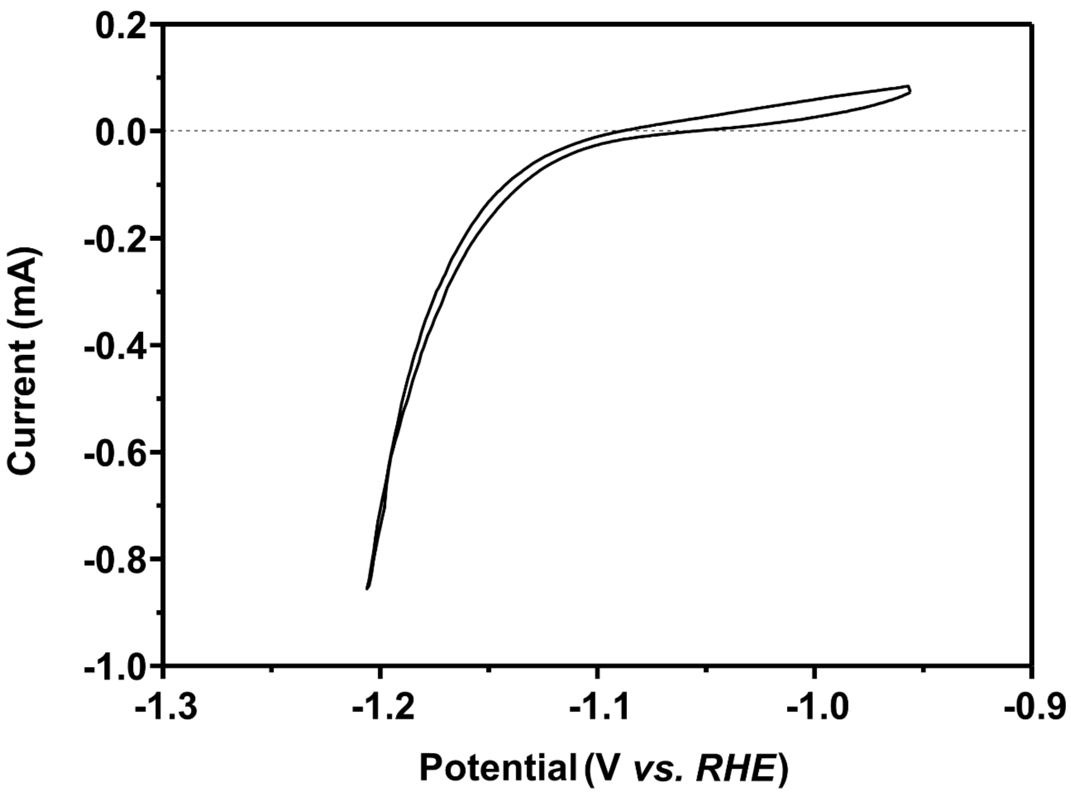

Fig. S13. Calibration of the saturated calomel electrode in 1 M KOH with an H_2 atmosphere. The potential at 0 mA is 1.0725 V ($\frac{1.089+1.057}{2}$). Therefore, the equation for transferring SCE to RHE potential was confirmed to be $E(RHE) = E(SCE) + 1.0725\,V$

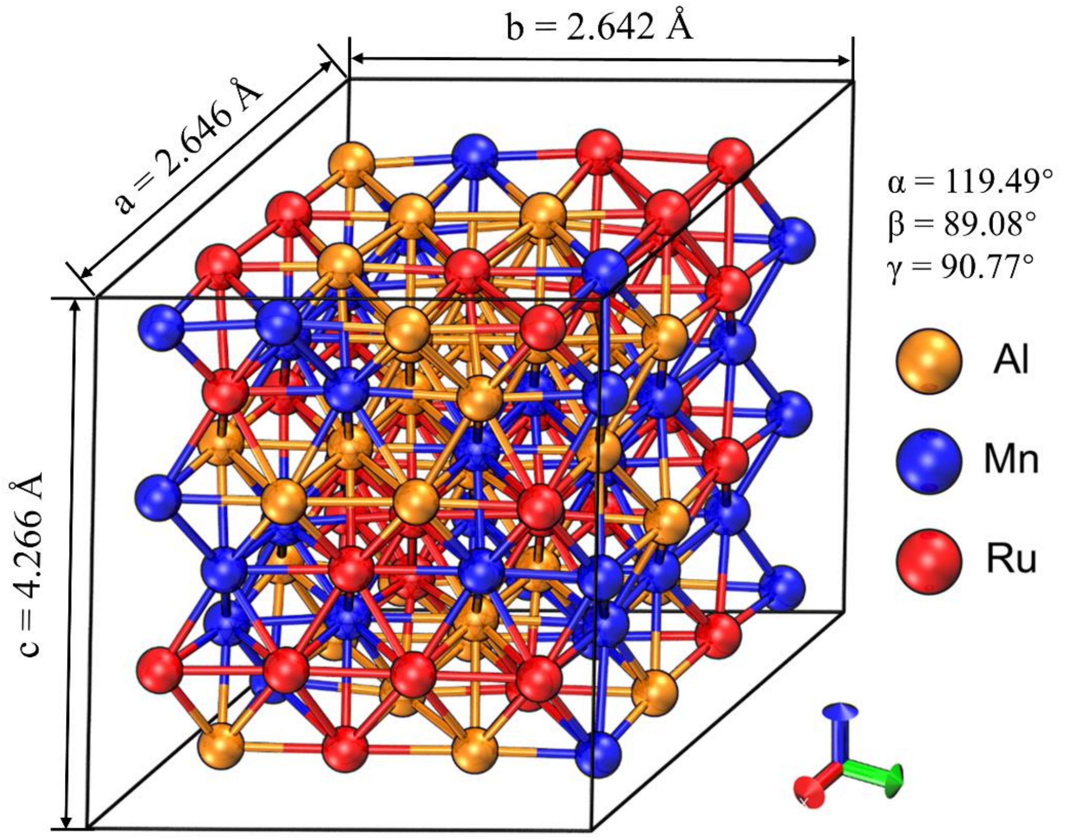

Fig. S14. Optimized unit cell of AlMnRu using DFT. Lattice parameters: a = 2.646 Å, b = 2.642 Å, c = 4.266 Å, a = 119.49 °, b = 89.08 °, g = 90.77 °.
